# Unveiling the perceptions of medical and allied health students towards cadaveric dissection and virtual resources in anatomy education: a cross sectional study

**DOI:** 10.1186/s12909-025-07432-z

**Published:** 2025-06-03

**Authors:** Rana Elbeshbeishy, Rasha Salama, BK Manjunatha Goud, Rasha Babiker, Malay Jhancy, Nour Hamed, Farah Oraby, Tarig Merghani

**Affiliations:** 1 Department of Anatomy, RAK College of Medical Sciences, RAK Medical and Health Sciences University, P.O. Box 11172, Ras Al Khaimah, 11172 United Arab Emirates; 2 Department of Community Medicine, RAK College of Medical Sciences, RAK Medical and Health Sciences University, Ras Al Khaimah, 11172 United Arab Emirates; 3 Department of Public Health and Community Medicine, Kasr El Aini Faculty of Medicine, Cairo University, Giza, Egypt; 4 Department of Biochemistry, RAKCOMS, RAK Medical and Health Sciences University, Ras Al Khaimah, 11172 United Arab Emirates; 5 Department of Physiology, RAK College of Medical Sciences, RAK Medical and Health Sciences University, Ras Al Khaimah, 11172 United Arab Emirates; 6 Department of Paediatric, RAK College of Medical Sciences, RAK Medical and Health Sciences University, Ras Al Khaimah, 11172 United Arab Emirates; 7 RAK College of Medical Sciences, RAK Medical and Health Sciences University, Ras Al Khaimah, 11172 United Arab Emirates

**Keywords:** Anatomy, Cadaveric dissection, Digital learning, Medical education, Student perceptions

## Abstract

**Background:**

Cadaveric dissection has long been a cornerstone of anatomy education, offering unparalleled hands-on experience that fosters both intellectual and emotional growth in medical students. It plays a crucial role in shaping professional identity while enhancing anatomical understanding. However, the emergence of digital platforms, including virtual reality (VR), augmented reality (AR), and 3D modeling, presents new opportunities to transform anatomy education.

**Objectives:**

This study explores the perceptions of medical and health sciences students at Ras Al Khaimah Medical and Health Sciences University (RAKMHSU) regarding the integration of digital tools alongside traditional cadaveric dissection. By comparing attitudes toward these approaches, the study aims to identify strategies like using 3D models, animations, and interactive apps to familiarize students with anatomical structures before entering the dissection lab for harmonizing traditional and digital learning methods to optimize anatomy education.

**Methods:**

This cross-sectional study was conducted over seven months using an anonymous, structured 20-item questionnaire administered to 454 students from various health disciplines at Ras Al Khaimah Medical and Health Sciences University. A convenience sampling method was used. The questionnaire assessed sociodemographic data, attitudes toward cadaveric dissection, its significance in practical learning, comparisons with digital resources, and its role in professional development. Institutional approval was obtained, and informed written consent was secured from all participants before the study commenced.

**Results:**

Medical students expressed significantly stronger support for cadaveric dissection compared to allied health students, particularly regarding emotional engagement (*p* < 0.05), perceived educational value (*p* < 0.001), and comfort with handling cadavers (*p* < 0.001). While both groups acknowledged the benefits of digital resources, medical students reported greater improvements in visualization, confidence, and interactive learning (*p* < 0.01).

**Conclusion:**

Cadaveric dissection remains an essential pillar of anatomy education, offering irreplaceable benefits in anatomical comprehension and psychomotor skill development. This study highlights its role in fostering professional attributes while demonstrating that digital platforms serve as valuable complementary tools. Rather than replacing cadaveric dissection, these digital innovations enhance learning by creating a synergistic educational environment.

## Background

Human anatomy is an ancient discipline derived from the Greek word anatome, meaning dissection [1]. The way anatomy is taught has been evolving over the centuries, but cadaveric dissection has remained integral to medical education. The world of anatomical study is thus an endless study wherein the medical as well as allied health sciences students begin their academic journey by engaging with the complexities of human anatomy, while balancing scientific understanding with ethical considerations and emotional resilience. Cadaveric dissection has remained for centuries a mainstay of anatomical education, allowing students the tactile and profound experiences of exploring the human body [2]. The traditional course provides students with opportunities to touch and examine real human tissues, gain insight into the intricacy of biological systems, and respect the body of donated humans [3]. Besides its educational merits, cadaver-based learning is a transformative process marking the passage of students from laypeople to medical professionals enshrining the core values of empathy, respect, and professionalism [4].

Recent trends within the academic field of teaching anatomy are being complemented by innovations in digital technology as credible pedagogical tools. Combined educational experiences, like virtual reality (VR), augmented reality (AR) or advanced 3D-modelling, offer students interactive, visually engaging, and flexible learning opportunities in which they can revisit and investigate complex anatomical concepts at their own timing and location without limitation [5].

By definition, VR creates a fully immersive, computer-generated environment that replaces the real world. Users wear headsets (like Oculus or HTC Vive) to explore and interact with a virtual space. Students can “enter” a virtual body to study structures in 360 degrees, enhancing spatial understanding and allowing repeated, safe exploration of anatomy. Whereas AR overlays digital elements (like images or 3D models) onto the real world using devices such as smartphones, tablets, or AR headsets (e.g., Microsoft HoloLens), 3D modeling involves creating digital representations of objects that can be viewed, rotated, and manipulated on screens or in VR/AR platforms. AR lets students view and manipulate anatomical models in real-time over cadavers or mannequins, aiding correlation between digital and real structures. High-resolution 3D anatomical models (e.g., bones, organs) are used in apps like Complete Anatomy or Visible Body for detailed study before, during, or after dissections [5, 6].

The incorporation of these innovative technologies into anatomical curricula has opened new paths for studying human anatomy, creating an alternative or complementary experience to traditional cadaveric dissection [6]. The implementation of these advancements has extraordinary potential in demystifying student comprehension while resolving some challenges of cadaveric learning, including limited specimen resources and emotional burdens for certain learners [7, 8]. The usefulness of cadaveric dissection has been widely established within anatomical education, though the validity of technology-based alternatives is now in academic contention. Some studies indicate that, when student learning is primarily evaluated based upon performance on examinations, technology-based modalities yield academically valid outputs equivalent to those achieved using traditional cadaveric dissection [2, 9, 10, 11].

Hands-on dissection is taken to be one of the sacred necessities enshrined in the heart of every medical student’s education by every professional anatomical association in the world for a comprehensive understanding of the structure and functioning of the human body. It also forms an important medium for professional identity formation by allowing the student to internalize their attitudes toward death, caregiving, and the human condition [12]. Even if replication of the tactile stroke will never be given to these technologies, they should accomplish a lot in favor of accessibility, convenience, and accommodating individual paths of learning. Nevertheless, the evolution of medical education has prompted the emergence of new technologies and pedagogies that conceptually complement, if not replace, cadaveric dissection.

Research has pointed out that experiences of emotions and self-reflection derived from cadaveric dissection are fundamental in the formation of the students’ professional identities [13, 14]. Thus, medical educators have sought to transform students’ emotional experiences into a constructive education [15].

Cadaveric dissection has long been regarded as a cornerstone of anatomical education, offering hands-on experience and a deep understanding of human structure. However, in recent years, it has faced increasing skepticism in many medical schools due to the adoption of newer teaching methods such as virtual simulations, 3D models, and interactive digital platforms. A few of such institutions have minimized dissection, while others abandoned the practice altogether to favor newer alternative pedagogical methods that were emerging in response to technological improvement and logistical concerns [16].

The current study aims to explore the perceptions, and attitudes of students toward cadaveric dissection, as well as its effectiveness in learning anatomy and developing clinical skills. Additionally, the study examines students’ perspectives on the integration of virtual resources in anatomy education. By considering students’ views and opinions, this initiative aims to merge theories of tactile experience with virtual instruction toward a common cause of transforming anatomy education. In this respect, the premise of this study is to allow new digital solutions to work side by side with the classical cadaveric dissections.

## Methods

### Study design and setting

A cross-sectional survey study was implemented over a seven-month duration at RAKMHSU colleges, namely medical, pharmacy, dental, and nursing colleges to assess the perception and attitude of students toward cadaveric dissection, in addition to its effectiveness in learning anatomy and developing clinical skills.

### Study population and sample size

A convenience sampling method was used to recruit students from RAKMHSU. The study included 454 students from the medical (*n* = 249), dental (*n* = 97), nursing (*n* = 52), and pharmacy (*n* = 52) colleges who participated in cadaveric dissection during the 2023–2024 academic year, representing various academic years, genders, and nationalities. This approach was chosen for its practicality in accessing students available during the study period. Exclusion criteria included postgraduate students, undergraduate students not enrolled in anatomy courses, and those who did not provide consent.

At RAK Medical and Health Sciences University (RAKMHSU), anatomy is taught using a systemic approach across all programs, with variations in depth and practical exposure tailored to the specific requirements of each discipline. Medical students (MBBS) receive the most extensive anatomy training, with approximately 2 h per week, including hands-on cadaveric dissection of all major body regions such as the thorax, abdomen, pelvis, limbs, and head and neck. Dental students (BDS) study anatomy, spending about 2 h per week, with limited dissection focused primarily on the head and neck region. Whereas, Nursing (BSc Nursing) and Pharmacy (BPharm) students receive anatomy instruction, with 1–2 h per week respectively, dedicated to practical sessions using models, virtual tools, and prosected specimens. While they are exposed to cadaveric dissection during interprofessional education activities, they do not participate in hands-on dissection. Furthermore, during the academic year 2023-24, all students had access to university-provided virtual anatomy resources (e.g., Complete Anatomy and 3D organ visualization tools), although VR headsets or immersive virtual reality environments were not used as part of formal instruction.

### Ethical considerations

Institutional approval was obtained before the study. Students were informed about the study objectives and procedures during free times in class by the investigators. Written informed consent was obtained from all participants for their voluntary participation. Data was collected without any identifying information to ensure confidentiality and anonymity.

### Data collection method

A structured questionnaire was developed through a multi-step process to ensure content validity and reliability, with items derived from a thorough review of existing literature on anatomy education and digital learning tools [17, 18, 19]. A panel of three experts reviewed the draft questionnaire, assessing its clarity, relevance, and comprehensiveness, and their feedback guided necessary revisions to ensure appropriateness for the study population. A pilot study was then conducted with a total of 15 student representatives participated, selected to ensure a diverse representation across the health sciences programs at RAKMHSU. Among them, 6 were medical (MBBS) students, 3 were dental (BDS) students, 3 were from the pharmacy (BPharm), and 3 from the nursing (BSc Nursing) programs. The majority of participants were in their first or second year of study; their responses and feedback were collected but not included in the final analysis. The questionnaire was refined based on this feedback, with reliability assessed using Cronbach’s alpha. Items with a Cronbach’s alpha value above 0.7 were retained, while those below this threshold were re-evaluated for modification or exclusion. The final questionnaire consisted of six sections: demographic information, perceptions of experiences with cadaveric dissection, attitudes toward cadaveric dissection, effectiveness of cadaveric dissection in learning anatomy and developing clinical skills, and attitudes toward virtual resources in anatomy education. Data were collected from 454 students who participated in cadaveric dissection sessions, with surveys distributed either during these sessions or in classrooms, depending on the students’ academic year. Clear instructions were provided to ensure proper understanding, and participants were given sufficient time to complete the survey. All responses were collected anonymously to maintain privacy and confidentiality.

### Data management and analysis

The data collected were then entered into Microsoft Excel for initial processing, after which coding was done, and then imported to IBM SPSS Statistics version 29. Data summary was done using descriptive statistics like frequency distributions and percentage calculations. Chi-square test was used to find the association between categorical variables. A *P*-value < 0.05 was held as a statistically significant.

## Results

The study included 454 students with a mean age of 20.5 ± 3.3 years, of whom 231 (51%) were male. A total of 249 students (54.8%) were from the College of Medicine, while the remaining participants were from Allied Health Sciences programs, including 97 (21.4%) from Dentistry, 54 (11.9%) from Nursing, and 54 (11.9%) from Pharmacy. Specifically, 367 students (80.9%) were in the basic science years, mainly the first and second years (298), while 87 students (19.1%) were in the clinical years (Year 4 and above).

**Fig. 1:**
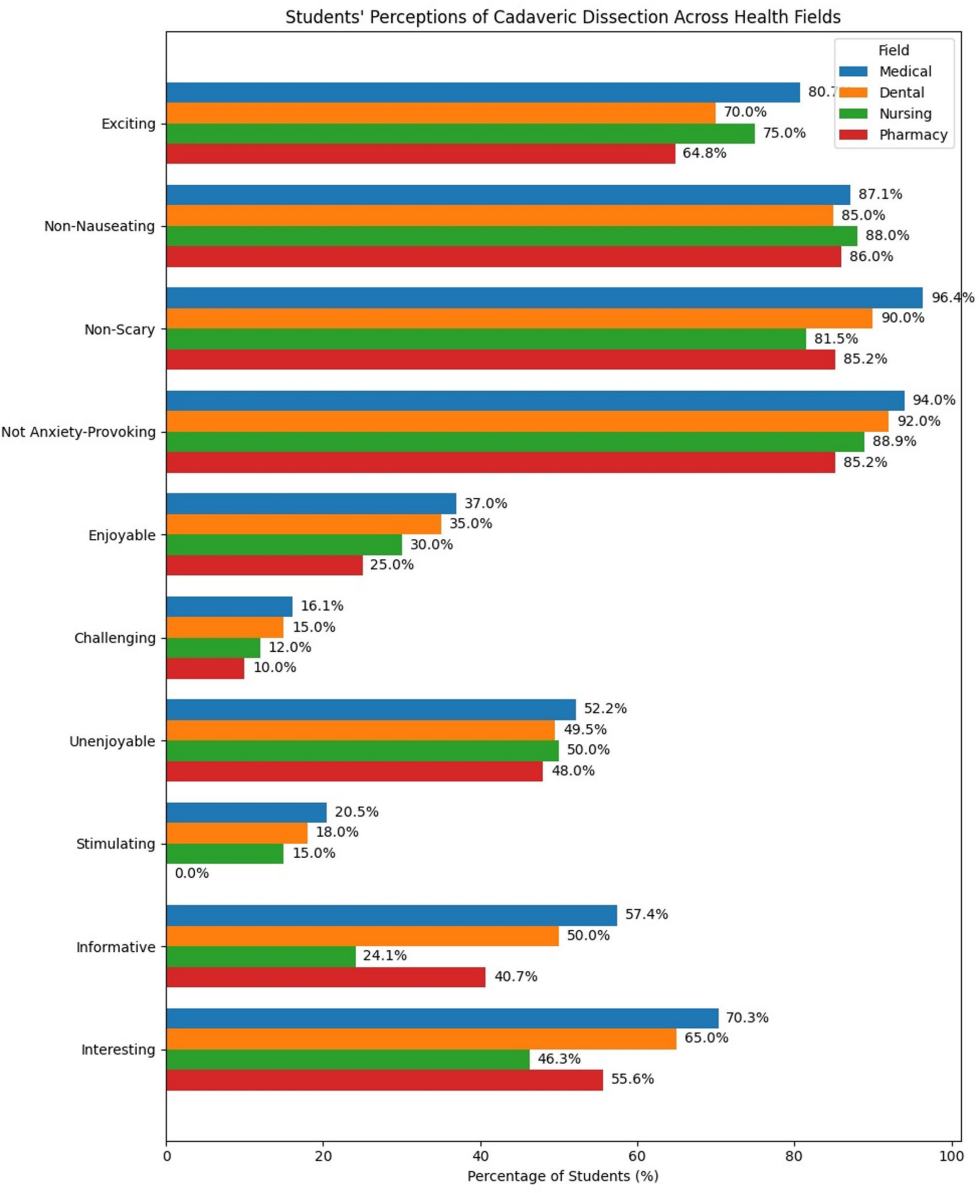
Students’ perceptions of cadaveric dissection experiences by college (*n* = 454)

Figure 1 presents students’ perceptions across different health-related fields (Medical, Dental, Nursing, and Pharmacy) regarding their experiences with cadaveric dissection. Medical students reported high levels of behavioral and cognitive engagement, that is defined as the degree of attention, curiosity, interest, motivation, and active involvement that students show when they are learning or being taught. It reflects how invested they are in their education and how willing they are to participate in learning activities. Medical students reported highly positive perceptions, with 80.7% (201 students) finding the experience exciting, a significantly higher proportion than Pharmacy students, of whom 64.8% (35 students) reported the same (*p* = 0.047). Most students across fields found dissection non-nauseating, with 87.1% (217 students) of medical students, and similarly high percentages in other fields (*p* = 0.834). However, 96.4% (240 students) of medical students found the experience non-scary, compared to 81.5% (44 students) in Nursing and 85.2% (46 students) in Pharmacy, a statistically significant difference (*p* = 0.001).

Regarding emotional impact, most students (96–98%) did not find the experience painful, depressing, or unbearable, with no significant variation across fields. A majority (85–89%) did not report feeling anxiety, though Nursing (11.1%, 6 students) and Pharmacy (14.8%, 8 students) students had higher anxiety-provoking responses than medical students (6.0%, 15 students, *p* = 0.041). A total of 168 students (37%) found the dissection enjoyable, while 73 students (16.1%) found it challenging. Perceptions of enjoyment and challenge did not significantly differ; however, 52.2% (130 students) of medical students found the dissection unenjoyable, which was slightly higher than the 49.5% (48 students) of dental students (*p* = 0.052). Additionally, 20.5% (56 students) of medical students found the experience stimulating, significantly more than the 0.0% (0 students) of pharmacy students (*p* = 0.001).

Interestingly, a higher percentage of medical students (57.4%, 143 students) found the experience informative compared to Nursing (24.1%, 13 students) and Pharmacy (40.7%, 22 students) students, with this difference reaching statistical significance (*p* < 0.001). Lastly, 70.3% (175 students) of medical students found it more interesting than Nursing (46.3%, 25 students) and Pharmacy (55.6%, 30 students) students (*p* = 0.001).

Table 1 highlights students’ attitudes toward cadaveric dissection. Among the groups, medical students showed the highest agreement that cadaveric dissection sessions should be increased, with 122 out of 249 (49.0%) strongly agreeing, while allied health students had the lowest agreement, with 39 out of 205 (19.0%) strongly agreeing (*p* < 0.001). Regarding cadaveric dissection as conducive to learning, 129 medical students (51.8%) strongly agreed—the highest level—compared to 35 allied health students (17.1%) (*p* < 0.001).

**Table 1:** Students’ perceptions of cadaveric dissection by college

Perceptions	Response	MEDICAL (249)	ALLIED (205)	*P*-value
Do you think that we have to increase cadaveric dissection sessions during practical environment?	Strongly Disagree	4(1.6%)	1(0.5%)	**48.871**, **< 0.001**
	Disagree	3(1.2%)	3(1.5%)	
	Neutral	33(13.3%)	57(27.8%)	
	Agree	87(34.9%)	105(51.2%)	
	Strongly Agree	122(49.0%)	39(19.0%)	
Do you think that cadaveric dissection favors conducive learning?	Strongly Disagree	4(1.6%)	5(2.4%)	**66.602**, **< 0.001**
	Disagree	5(2.0%)	4(2.0%)	
	Neutral	22(8.8%)	56(27.3%)	
	Agree	89(35.7%)	105(51.2%)	
	Strongly Agree	129(51.8%)	35(17.1%)	
Did you have any fear of touching the cadaver directly?	Strongly Disagree	118(47.4%)	35(17.1%)	**73.519**, **< 0.001**
	Disagree	71(28.5%)	42(20.5%)	
	Neutral	35(14.1%)	73(35.6%)	
	Agree	18(7.2%)	40(19.5%)	
	Strongly Agree	7(2.8%)	15(7.3%)	
Did you experience emotional shock upon first being exposed to the cadaver?	Strongly Disagree	103(41.4%)	30(14.6%)	**49.874**, **< 0.001**
	Disagree	66(26.5%)	51(24.9%)	
	Neutral	38(15.3%)	70(34.1%)	
	Agree	33(13.3%)	46(22.4%)	
	Strongly Agree	9(3.6%)	8(3.9%)	
Did you ever experience any kind heartedness and regard for the cadaver you dissected?	Strongly Disagree	41(16.5%)	20(9.8%)	**10.068**, **0.039**
	Disagree	48(19.3%)	50(24.4%)	
	Neutral	84(33.7%)	88(42.9%)	
	Agree	62(24.9%)	39(19.0%)	
	Strongly Agree	14(5.6%)	8(3.9%)	
Did you experience any anxiety or stress at the beginning of the dissection?	Strongly Disagree	70(28.1%)	27(13.2%)	**18.384**, **0.001**
	Disagree	77(30.9%)	60(29.3%)	
	Neutral	54(21.7%)	65(31.7%)	
	Agree	41(16.5%)	46(22.4%)	
	Strongly Agree	7(2.8%)	7(3.4%)	
Did you feel haunted after the day of dissection lab?	Strongly Disagree	134(53.8%)	53(25.9%)	**44.638**, **< 0.001**
	Disagree	69(27.7%)	70(34.1%)	
	Neutral	31(12.4%)	52(25.4%)	
	Agree	11(4.4%)	28(13.7%)	
	Strongly Agree	4(1.6%)	2(1.0%)	
Did you get irritated with the smell of formalin?	Strongly Disagree	19(7.6%)	9(4.4%)	7.996, 0.092
	Disagree	38(15.3%)	26(12.7%)	
	Neutral	66(26.5%)	78(38.0%)	
	Agree	95(38.2%)	68(33.2%)	
	Strongly Agree	31(12.4%)	24(11.7%)	
I often speak with others about my experience in the dissection lab	Strongly Disagree	7(2.8%)	3(1.5%)	**33.893**, **< 0.001**

When asked about fear of touching the cadaver, medical students showed the least fear, with 118 (47.4%) strongly disagreeing, compared to 35 (17.1%) among allied health students (*p* < 0.001). Similarly, emotional shock upon first exposure was less common among medical students; 103(41.4%) strongly disagreed, versus 30(14.6%) in allied health students (*p* < 0.001). On feeling haunted after dissection, 134 medical students (53.8%) strongly disagreed compared to 53 allied health students (25.9%) (*p* < 0.001). When asked whether they often speak about their dissection lab experience, 69 medical students (27.7%) strongly agreed, while only 19 allied health students (9.3%) did so (*p* < 0.001).

**Fig. 2:**
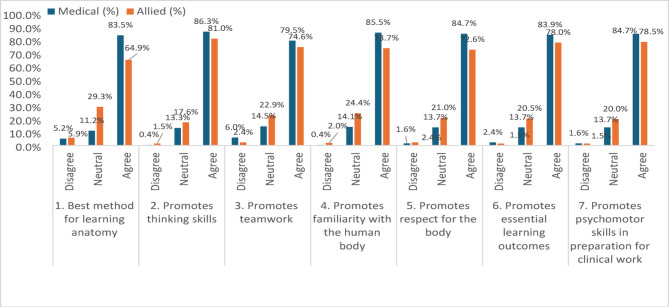
Attitudes of Medical and Allied Health Students Towards the effect of Cadaveric Dissection in Learning Anatomy and Developing Clinical Skills

Attitudes toward cadaveric dissection as a learning tool (Fig. 2) also varied: 126 medical students (50.6%) strongly agreed it is the best method for learning anatomy, compared to 44 allied health students (21.5%) (*p* < 0.001). For helping students develop familiarity with the human body, 117 medical students (47.0%) strongly agreed versus 64 allied health students (31.2%) (*p* = 0.001). Regarding the role of dissection in supporting essential learning outcomes, 121 medical students (48.6%) strongly agreed, compared to 66 allied health students (32.2%) (*p* = 0.003).

However, no significant differences were observed between the groups in terms of promoting **orderly thinking** skills, with 121 medical students (48.6%) and 97 allied health students (47.3%) agreeing or strongly agreeing (*p* = 0.337); teamwork, with 198 medical students (79.5%) and 153 allied health students (74.6%) agreeing or strongly agreeing (*p* = 0.051); or respect for the human body, with 211 medical students (84.8%) and 157 allied health students (76.6%) agreeing or strongly agreeing (*p* = 0.084).

Table 2 shows the attitudes toward the use of university virtual resources in anatomy education among medical and allied health students. A majority of both groups agreed or strongly agreed that virtual resources enhance understanding of anatomical structures, with 148 medical students (59.5%) and 125 allied health students (60.9%) expressing positive views; this difference was not statistically significant (*p* = 0.228). Additionally, both groups acknowledged the flexibility offered by virtual resources, allowing students to study at their own pace, with 185 medical students (74.3%) and 130 allied health students (63.4%) agreeing or strongly agreeing, with no significant difference (*p* = 0.116).

**Table 2:** Attitudes towards the use of virtual resources in anatomy education among medical and allied health students

Do you think that virtual resources, such as 3D anatomy applications, virtual dissection software, metaverse AI… etc. enhance understanding of anatomical structures compared to traditional methods?	Strongly Disagree	6(2.4%)	1(0.5%)	5.632, 0.228
	Disagree	28(11.2%)	16(7.8%)	
	Neutral	67(26.9%)	63(30.7%)	
	Agree	96(38.6%)	88(42.9%)	
	Strongly Agree	52(20.9%)	37(18.0%)	
Do you believe that virtual resources offer greater flexibility in learning anatomy, allowing students to study at their own pace and convenience?	Strongly Disagree	1(0.4%)	1(0.5%)	7.397, 0.116
	Disagree	11(4.4%)	12(5.9%)	
	Neutral	52(20.9%)	62(30.2%)	
	Agree	128(51.4%)	97(47.3%)	
	Strongly Agree	57(22.9%)	33(16.1%)	
Do you think that digital platforms environments significantly amplify your in-depth visualization of anatomy of the human body over classic study techniques in terms of imagining different body compartments and layers?”	Strongly Disagree	1(0.4%)	0(0.0%)	**24.298**, **< 0.001**
	Disagree	15(6.0%)	7(3.4%)	
	Neutral	53(21.3%)	74(36.1%)	
	Agree	115(46.2%)	101(49.3%)	
	Strongly Agree	65(26.1%)	23(11.2%)	
Do you believe that integrating virtual resources into anatomy education improves students’ confidence in their anatomical knowledge and skills?”	Strongly Disagree	1(0.4%)	2(1.0%)	**13.888**, **0.008**
	Disagree	8(3.2%)	10(4.9%)	
	Neutral	47(18.9%)	63(30.7%)	
	Agree	125(50.2%)	96(46.8%)	
	Strongly Agree	68(27.3%)	34(16.6%)	
Do you think that virtual resources provide students with opportunities for more interactive and engaging learning experiences than traditional anatomy textbooks or lectures?	Strongly Disagree	3(1.2%)	3(1.5%)	**25.997**, **< 0.001**
	Disagree	17(6.8%)	4(2.0%)	
	Neutral	46(18.5%)	71(34.6%)	
	Agree	111(44.6%)	95(46.3%)	
	Strongly Agree	72(28.9%)	32(15.6%)	

However, significant differences emerged regarding the impact of digital platforms on in-depth anatomical visualization. A larger proportion of medical students (180 students; 72.3%) than allied health students (124 students; 60.5%) believed that virtual resources significantly enhanced their ability to visualize anatomy (*p* < 0.001). Similarly, 193 medical students (77.5%) agreed or strongly agreed that virtual tools improved their confidence in anatomical knowledge and skills, compared to 130 allied health students (63.4%) (*p* = 0.008). Lastly, 183 medical students (73.5%) viewed virtual resources as providing more interactive and engaging learning experiences than traditional methods, compared to 127 allied health students (61.9%) (*p* < 0.001).

## Discussion

The landscape of anatomy education is constantly evolving, with traditional methods like cadaveric dissection being complemented and sometimes challenged by new technologies such as virtual reality, augmented reality, computer-based simulations and 3D modeling. This study sought to understand how medical and allied health students perceive these different approaches, providing valuable insights into the changing dynamics of anatomical learning.

The results reveal a clear divergence in perceptions and attitudes towards cadaveric dissection and virtual resources between medical students and allied health students. This discrepancy highlights the influence of program-specific learning objectives and anticipated professional roles on students’ educational experiences. This aligns with the broader understanding that educational experiences are shaped by professional identity formation and anticipated future practice [20]. The “communities of practice” concept further supports this, suggesting that students’ learning is influenced by their future professional communities’ values, practices, and expectations [21].

Medical students consistently demonstrated a more positive and engaged experience with cadaveric dissection compared to their allied health counterparts (dental, nursing, and pharmacy). They reported higher levels of excitement, lower levels of anxiety, and a greater sense of the experience being informative, interesting, and stimulating. This is further supported by their stronger agreement that dissection should be increased, that it is conducive to learning, and that it is the best method for learning anatomy. They also expressed less fear of touching the cadaver and were more likely to discuss their lab experiences. These findings suggest that medical students, for whom a deep and detailed understanding of anatomy is crucial for invasive procedures and surgical interventions, perceive cadaveric dissection as a highly valuable and relevant learning tool. This strong endorsement of dissection by medical students is consistently reported in the literature, with many studies highlighting its role in developing spatial reasoning, tactile skills, and a holistic understanding of human anatomy [17, 22, 23, 24, 25]. Aziz et al. (2002), Smith and Lindwall (2025) and Winkelmann (2007) specifically emphasized the “embodied” nature of learning through dissection, arguing that the physical interaction with the cadaver provides a unique sensory experience that cannot be replicated by other methods [10, 23, 24]. Additionally, studies have reported that students faced stress and anxiety at the beginning of their first exposure to dissection labs [18, 26] which contradicts the current study results. The contradictory results may be attributable to the study participants who were female students in one study [18], and the second study was conducted on 167 medical students within 6 weeks of their exposure to dissection rooms [26]. In contrast, allied health students, whose future roles may involve less direct physical manipulation of the human body, displayed a more ambivalent attitude towards dissection. While they generally did not find the experience nauseating, depressing, or unbearable, they reported higher levels of anxiety and a lower perceived value of dissection in terms of its informativeness and interest. This difference likely stems from the varying emphasis placed on detailed anatomical knowledge in their respective curricula. For instance, while a pharmacist needs to understand the physiological effects of drugs on the body, the level of detailed anatomical knowledge required might not be as extensive as that needed by a surgeon. Similarly, nurses, while requiring a solid understanding of anatomy, may prioritize other skills, such as patient care and clinical assessment [27, 28]. This aligns with recent research findings that suggest the perceived relevance of dissection is linked to its perceived utility in future practice [29]. However, it is important to note that some studies have shown that allied health students do value dissection for developing spatial awareness and contextualizing anatomical knowledge within their professional context, indicating that a tailored approach to dissection within allied health curricula could be beneficial [30]. Recent research by Bergen et al. (2024) explored the perspectives of physiotherapy students on cadaveric dissection and found that while they valued the experience for enhancing their understanding of musculoskeletal anatomy, they also suggested integrating more clinically relevant scenarios into the dissection sessions to improve its perceived relevance to their future practice [31].

Interestingly, while medical students strongly endorsed dissection as the best method for learning anatomy and for developing familiarity with the human body, there were no significant differences between the groups regarding the promotion of orderly thinking skills, teamwork, and respect for the human body. This suggests that while dissection may be perceived differently in terms of its direct anatomical teaching value, its potential for fostering broader professional skills is recognized across disciplines. This finding contrasts somewhat with research that suggests cadaveric dissection uniquely contributes to the development of professional attributes like empathy and ethical awareness in medical students [32]. This discrepancy could be due to differences in how these attributes are measured or the specific learning activities incorporated into the dissection experience. A recent study by Ong et al. (2023) examined the effects of cadaveric dissection on medical students’ professional development, specifically focusing on the development of empathy and respect. The researchers found that when structured activities were implemented to encourage students to reflect on their experiences—both during the dissection process (e.g., through guided discussions) and afterward (e.g., through reflective writing or debriefing sessions)—there was a noticeable improvement in these crucial professional qualities [4].

While both medical and allied health students generally agreed on the enhancing effect of virtual resources on understanding anatomical structures and the flexibility they offer, significant differences emerged regarding their impact on in-depth visualization, confidence in anatomical knowledge, and the perception of interactivity and engagement. Medical students expressed a significantly stronger belief that virtual resources enhanced their visualization skills, boosted their confidence, and provided a more engaging learning experience. This could be attributed to medical students utilizing virtual resources as a supplementary tool to their extensive hands-on experience with cadaveric dissection, allowing them to reinforce and consolidate their learning in a different modality. For allied health students, who have less direct experience with donated human bodies, virtual resources might be seen as a primary learning tool, but perhaps not as effectively replacing the tactile and three-dimensional experience of dissection in fostering deep visualization and confidence. This resonates with studies that suggest virtual resources can be highly effective for learning anatomy, particularly in terms of spatial understanding and allowing for repeated practice [33, 34], but that they may not fully replicate the complex learning experience provided by dissection. Minouei et al. (2024), in their systematic review, mentioned that the use of virtual reality, medical anatomy improved spatial ability and anatomical knowledge, particularly when used as a supplement to traditional teaching methods [33]. Virtual reality (VR) in anatomy education is a technology-based method that immerses students in a fully digital, three-dimensional environment where they can explore and interact with realistic models of the human body. Using VR headsets and controllers.

### Limitation of the study

This study has a few limitations that should be acknowledged. First, the study sample was drawn from a single institution, which limits the generalizability of the findings. Students at other institutions may experience different curricula, have access to varying resources, and represent different demographic backgrounds, all of which could influence their perceptions and learning experiences. Therefore, further research involving multiple institutions is recommended to enhance the generalizability of the findings. Second, the study relies on self-reported data collected through surveys or questionnaires. This methodology is susceptible to response bias, as students may provide socially desirable answers rather than reflecting their true feelings, or they may not accurately recall their experiences or perceptions. Finally, the study likely employed a cross-sectional design, capturing data at a single point in time. This design precludes the establishment of causal relationships between the learning modalities (dissection and virtual resources) and student outcomes. Longitudinal studies are necessary to assess the long-term impact of these methods on knowledge retention, the development of clinical skills, and professional development.

### Recommendations

For medical education, cadaveric dissection should remain a core component of the curriculum due to its unique contribution to developing tactile skills, spatial reasoning, and a holistic understanding of human anatomy, which cannot be easily replicated by other methods [35]. However, integrating virtual resources as supplementary tools can further enhance learning by providing opportunities for review, self-assessment, and exploration of complex anatomical structures in different modalities. While dissection teaches anatomical knowledge, it also provides a powerful opportunity to cultivate a deeper appreciation for the human body and the individuals who donated their bodies for education. By guiding students to reflect on their emotional responses, ethical considerations, and the human aspect of cadaver-based learning, educators can promote the development of compassionate and respectful attitudes—qualities essential for future healthcare professionals. One practical example of this is a post-dissection reflective journal. After a dissection session, students are asked to write about their experiences. These reflections can be later discussed in small groups or with faculty to deepen insight and promote professional growth. For allied health education, while cadaveric dissection may still offer some benefits, particularly in developing spatial awareness, its perceived value and relevance are lower. Therefore, curricula should prioritize alternative pedagogical approaches that align more closely with their specific learning objectives and future professional roles. These approaches may include the strategic integration of virtual resources such as virtual reality, augmented reality, and 3D modeling software to provide engaging and interactive learning experiences for visualizing complex anatomical structures and processes; prosection viewing, which offers a valuable visual and tactile experience without the emotional and practical challenges of full dissection; high-fidelity simulations that incorporate anatomical principles to help students apply their knowledge in realistic scenarios. for example, use of virtual dissection tables like the Anatomage Table. This life-sized, interactive touchscreen allows students to explore full-body 3D human anatomy models derived from real cadaver scan; case-based learning using real-world clinical cases to provide context and demonstrate relevance to patient care; and tailored dissection experiences focusing on anatomical regions and concepts most relevant to the specific allied health profession, such as a focus on musculoskeletal anatomy for physiotherapy students. Future research should focus on investigating the optimal combination of cadaveric dissection and affordable virtual resources for different health professions, exploring the long-term impact of these learning modalities on clinical practice and patient outcomes, conducting qualitative studies to gain deeper insights into students’ learning experiences and perceptions, evaluating the effectiveness of new and emerging technologies such as haptic feedback and advanced visualization techniques, and developing standardized assessment tools to measure the specific learning outcomes associated with different anatomical teaching methods.

## Conclusions

This study has illuminated distinct differences in perceptions and attitudes toward cadaveric dissection and virtual resources between medical students and allied health students. Medical students consistently expressed a strong preference for cadaveric dissection, perceiving it as a highly valuable tool for developing a deep understanding of anatomy, spatial reasoning, and essential professional skills. In contrast, allied health students demonstrated a more ambivalent attitude, likely due to varying curricular emphasis on detailed anatomical knowledge and the perceived relevance of dissection to their future practice. While both groups acknowledged the benefits of virtual resources in enhancing anatomical understanding and offering flexibility, medical students perceived a stronger impact on in-depth visualization, confidence, and engagement. These findings highlight the importance of tailoring educational strategies by integrating 3D models, animations, and interactive apps to familiarize students with anatomical structures before entering the dissection lab that address the specific needs and learning objectives of each discipline.

## Data Availability

The datasets used and/or analyzed during the current study are available from the corresponding author on reasonable request.

## Abbreviations

VR: Virtual reality
AR: Augmented reality
RAKMHSU: Ras Al Khaimah Medical and Health Sciences University
